# Retraction: Novel biocompatible core/shell Fe_3_O_4_@NFC@Co(ii) as a new catalyst in a multicomponent reaction: an efficient and sustainable methodology and novel reusable material for one-pot synthesis of 4*H*-pyran and pyranopyrazole in aqueous media

**DOI:** 10.1039/d3ra90127k

**Published:** 2024-01-05

**Authors:** Pouya Ghamari Kargar, Ghodsieh Bagherzade, Hossein Eshghi

**Affiliations:** a Department of Chemistry, Faculty of Sciences, University of Birjand Birjand 97175-615 Iran gbagherzade@gmail.com bagherzadeh@birjand.ac.ir +98 56 32345192 +98 56 32345192; b Department of Chemistry, Faculty of Science, Ferdowsi University of Mashhad Mashhad Iran

## Abstract

Retraction of ‘Novel biocompatible core/shell Fe_3_O_4_@NFC@Co(ii) as a new catalyst in a multicomponent reaction: an efficient and sustainable methodology and novel reusable material for one-pot synthesis of 4*H*-pyran and pyranopyrazole in aqueous media’ by Pouya Ghamari Kargar *et al.*, *RSC Adv.*, 2020, **10**, 37086–37097, https://doi.org/10.1039/D0RA04698A.

The Royal Society of Chemistry, with the agreement of the named author, hereby wholly retracts this *RSC Advances* article due to concerns with the reliability of the data.

The XRD patterns in Fig. 2a contain repeating sections.

The authors provided the raw XRD data for Fe_3_O_4_ in Fig. 2a of this article and it was found to be identical in a number of different regions to the raw data provided by the authors for CuO in Fig. 4b of ref. 1 and Fig. 3 of ref. 2.

The authors have stated that they outsourced the XRD data collection to an external company.

Given the significance of these concerns, the findings presented in this paper are no longer reliable.

The authors were informed about the retraction of the article. Pouya Ghamari Kargar and Ghodsieh Bagherzade have not agreed with the decision.

Signed: Hossein Eshghi

Date: 13^th^ December 2023

Retraction endorsed by Laura Fisher, Executive Editor, *RSC Advances*

## Supplementary Material
